# Cost-Effectiveness Analysis of Topiramate versus Phenobarbital in the Treatment of Children with Febrile Seizure

**Published:** 2019

**Authors:** Hamid NEMATI, Hamid TALEBIANPOUR, Farhad LOTFI, Nazanin Zahra SEPEHRI, Khosro KESHAVARZ

**Affiliations:** 1 Neuroscience Research Center, Shiraz University of Medical Sciences, Shiraz, Iran.; 2 Department of Health Care Management, School of Management and Medical Information, Shiraz University of Medical Science, Shiraz, Iran.; 3 Health Human Resources Research Center, School of Management & Medical Informatics, Shiraz University of Medical Sciences, Shiraz, Iran.

**Keywords:** Febrile seizure, Topiramate, Phenobarbital, Cost-effectiveness, Cost-utility

## Abstract

**Objectives:**

Febrile seizure is common disorder in childhood, with a prevalence of 2% to 5%. There are many drugs for treatment of this disease; however, the most common prescribed medication in Iran is phenobarbital that is cheap, but it has many side effects. We aimed to compare the cost-effectiveness of topiramate versus phenobarbital in patients with febrile seizure in the south of Iran.

**Materials & Methods:**

This econometric cost-effectiveness and cost-utility study were conducted on 91 patients with febrile seizure to assess two strategies of oral drug therapy including phenobarbital and topiramate in 2016-2017. Of all, 51 patients were treated with phenobarbital and 40 patients received topiramate. We followed up the patients for six months, using a randomized and single-blinded approach. A decision tree model was used. The outcomes of the model included febrile seizure and utility. The study was conducted from the perspective of the community; therefore, direct and indirect costs were included in the study. Excel and Tree Age software (2011) were used to analyze the results.

**Results:**

Topiramate was cheaper and more effective than phenobarbital. In patients in the phenobarbital and topiramate groups, the mean costs were $740 and $674 per PPP, utility scores were 0.72 and 0.82, and febrile seizure without side effects were 0.3 and 0.6, respectively. Moreover, one-way sensitivity analysis confirmed the robustness of the results of the study.

**Conclusion:**

Topiramate in patients with febrile seizure is a fully cost-effective and cost-efficient strategy suggested as a better alternative for children with febrile seizure.

## Introduction

In childhood, febrile seizures are the most prevalent seizures (1). The prevalence of this disease in most parts of the world is 2% to 5%, and 1% to 6% of people with epilepsy have a history of febrile seizure (2). The disease is generally of two types, simple and complex. About 65% to 90% of the febrile seizures are simple (3). If one has a family history of febrile seizure, he/she will be 31% more likely than others to develop the disease, and family history seems to have a significant effect on febrile seizure (4). The prevalence of febrile seizure (in particular, complex febrile seizure) has increased over the past decade (5). 

No treatment is required for febrile seizure occurring once or twice, but it needs medications if repeated. Febrile seizure that lasts for more than five minutes requires treatment and medications, but in 30% of cases, the drugs have probable side effects (6). The occurrence of this disease can have a great impact on the parents and cause anxiety and tensions in the family (7). The pathophysiology of this disease is unknown and both genetic factors and environment can affect the disease (8). The prognosis of this disease is good and in some cases, it may progress to epilepsy (9, 10). The disease requires prolonged therapeutic courses and the treatment of patients with febrile seizure and epilepsy usually lasts for a long period. In addition, the drugs used for the treatment can cause many side effects and these side effects have a great impact on the patient's quality of life, and the patient has to incur lots of costs to treat these side effects (11). Therefore, it seems reasonable to avoid the repetition of febrile seizure as far as possible using safe methods (12).

Many drugs are prescribed for patients with febrile seizure and epilepsy, but because of the differences in the costs, effectiveness, and side effects, there are controversies over selecting the best drug to be prescribed (13). Phenobarbital is one of the drugs commonly prescribed in Iran. Phenobarbital is a drug used orally and intravenously to treat patients with this disease (14). Topiramate is another drug widely used in the world to treat local and general seizure (15). However, both topiramate and phenobarbital have side effects. Side effects of phenobarbital include behavioral problems, sleepiness, acne, and cognitive problems (16), and those of topiramate include weakness, sleepiness, lack of speech, depression, depression problems, hallucinations, imbalance, dizziness, numbness, headache, diarrhea, nausea, anorexia, speech impairment, sweating, kidney stones, infection, and fever (17-20). It seems reasonable to prevent the recurrence of febrile seizure, as far as possible, using safe methods. Nonetheless, there is no therapeutic regimen accepted by all experts. In addition, there are controversies over the efficacy and side effects of these drugs, and there are uncertainties over choosing the best drug to be prescribed by physicians (21).

Policymakers will never have enough money to do whatever they want, it is not enough to know all the existing interventions to solve a health problem; hence, they also need to be aware of the costs of interventions (22). Since there are different methods and medications for treating patients with this disease, it is very important to choose a method that is both more effective and less costly (23). 

Therefore, we aimed to assess the cost-effectiveness of phenobarbital and topiramate drugs in patients with febrile seizure in the south of Iran to identify the most cost-effective drug for patients with the disease.

## Materials & Methods

**Overview**

This cost-effectiveness study was conducted on 91 patients with febrile seizure referred to Nemazee & Dastgheib hospitals, and Imam Reza Clinic, Shiraz University of Medical Sciences, Shiraz, Iran, to assess two strategies of oral drug therapy including phenobarbital and topiramate in 2016-2017. Of all, 51 patients were treated with phenobarbital and 40 patients received topiramate. In order to collect the required data, using a randomized and single-blinded approach, we followed up the patients for six months. A decision tree model was used to estimate the economic and clinical outcomes. Data on costs were collected from the community viewpoint, and the lack of recurrence of febrile seizure and quality-adjusted life year (QALY) were set as the effectiveness outcomes. We used a form to collect data on costs and effectiveness; furthermore, EQ-5D questionnaire was used to estimate the utility scores. The results were presented in form of the Incremental Cost-Effectiveness Ratio (ICER). Moreover, one-way sensitivity analysis was performed to measure the uncertainty effects of the parameters in the model. TreeAge Pro 2011 and Excel 2016 software were used for the analysis of the collected data.

This Manuscript is extracted from MSc thesis that was funded and approved by Shiraz University of Medical Sciences with the ID number of 95-01-07-12635. In addition, it was approved by the Ethics Committee of Shiraz University of Medical Sciences with the ID number of IR.SUMS.REC.1396.S31. The informed consent was taken from all patients.

**Type of the study**

This study was an economic evaluation of the cost-effectiveness and cost-utility analysis, conducted as a single-blinded randomized controlled trial (RCT) in 2016-2017. Children under five years of age with febrile seizure in the south of the country were followed up for six months. The participants of the study were the patients with febrile seizure under five years of age referred to hospitals and clinics for the treatment of the disease by Mar 2017 and received one of the two drugs of topiramate or phenobarbital. The mean dose of topiramate and phenobarbital were 5-7 and 5 mg/kg/d in 2 divided doses; respectively. The number of patients receiving phenobarbital and topiramate was 51 and 40 persons, respectively. Our studied subjects included children who had more than two cases of complex febrile seizure or simple febrile seizure diagnosed by a pediatric neurologist. Considering the research objectives and community size, we used the census method to select the samples. Since topiramate was not familiar for these patients in Iran and was more commonly prescribed for patients with epilepsy, first, the drug was introduced to the parents of the patients and written consent forms were obtained from those who were willing to participate in the study. If not willing to participate in the study, they were allowed to withdraw from the study. The samples were divided into two groups, A and B. Then, a type of these drugs was prescribed for each group of patients. The patients were selected quite randomly through randomized block permutation design. The researcher made telephone calls to the parents of the patients, collected data, and completed the cost and effectiveness checklist for each drug.

**Inclusion and Exclusion Criteria**

The inclusion criteria were as follows: Age 6 months to 60 months, no history of afebrile seizure without fever, three times or more simple febrile seizure or complex seizure; lack of Central Nervous System (CNS) infection and without Electrolyte Imbalance. The exclusion criteria were change in the diagnosis of the disorder; resulting in continuation of another drug for the child; quitting treatment due to the occurrence of drug side effect.

**Clinical inputs**

To compare the efficacy of the drugs, we used the following clinical outcomes: lack of recurrent febrile seizure and utility. They were investigated through administering the drugs and following up the patients for six months. To determine the amount of success and failure of each drug in controlling the febrile seizure, the number of patients with febrile seizure was divided by the total number of patients in each group. Moreover, utility scores were obtained using EQ-5D questionnaire and patient interviews.

**Treatment Costs**

Data on direct medical costs were collected from outpatient medical records, as well as self-reports by the experts in the field. Data on direct non-medical costs and indirect costs were collected based on self-reports by the patients through face-to-face interviews or telephone calls.

The costs were calculated based on the tariffs in 2017 and converted to the international dollar (purchasing power parity) with an exchange rate of 1 dollar = 12032 Rials (24).

**Model structure**

**Figure 1:**
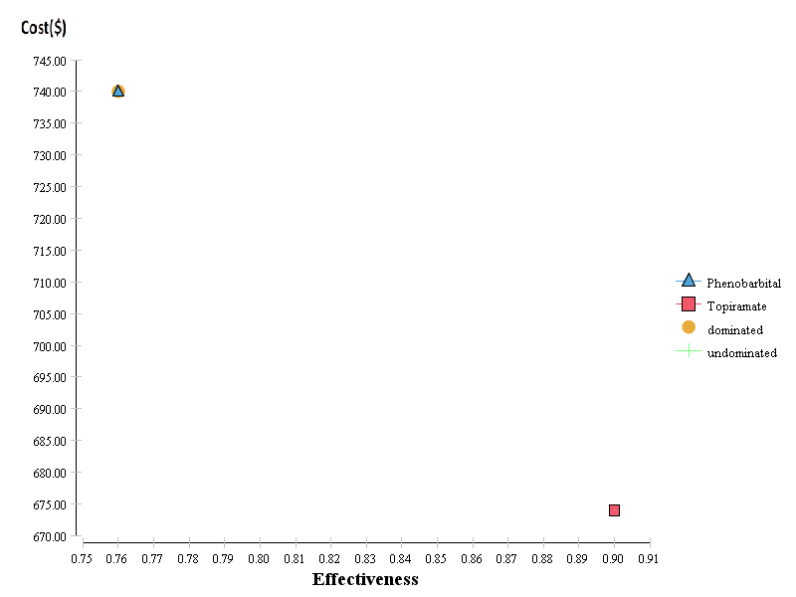
Cost-Effectiveness analysis of the use of phenobarbital and topiramate for treating patients less than five years of age with febrile seizure

Figure 1 shows the schematic diagram of decision tree model for taking phenobarbital versus topiramate.

Using decision tree model and TreeAge software, the two clinical options along with their cost of treatment, effectiveness, and complications of the disease were analyzed. This model assessed the recurrence of febrile seizure and utility in two groups of patients taking phenobarbital and topiramate. For each therapeutic strategy, the success and failure of the drugs and their side effects were plotted to choose the best treatment strategy.

**Cost-effectiveness analysis:** The model was designed in the Tree-age software and the extracted data were entered into the model; then, the costs, effectiveness, cost-effectiveness, and cost-utility were calculated for the two drugs and their ICER were estimated using the following equation.

$\mathrm{ICER}=\frac{\mathrm{CostA}-\mathrm{CostB}}{\mathrm{OutcomeA}-\mathrm{OutcomeB}}$

**Sensitivity analysis**

One-way sensitivity analysis was conducted to examine the effects of the uncertainty of the parameters on the results of the model. There was an attempt to modify the key parameters of the model, including the effectiveness and costs per drug, and the results are presented in the form of a tornado diagram. Because of the lack of a certain cost-effectiveness threshold in Iran, as the WHO has recommended for developing countries, the threshold for each QALY is one to three times more than the per capita gross domestic product (GDP); according to the Iranian Food and Drug Administration, it was about 113 million Rials in 2017; thus, its three-fold amount is about 339 million Rials.

## Results

Overall, 91 patients less than five years of age with febrile seizure were enrolled. Table 1 presents the descriptive results including the data on sex, education, occupation status, residence, and income, history of febrile seizure, type of febrile seizure, and type of drug used.

As shown in Table 2, in both phenobarbital and topiramate groups, the highest amount of cost was related to direct medical costs with mean values of $322.59 and $314.38 per pp, respectively, and the lowest amount of cost was related to indirect costs, with mean values of $283.32 and $257.23 per PPP, respectively. However, the mean total cost of treatment with phenobarbital ($739.3 per pp) was more than that of treatment with topiramate ($674 per PPP).

**Figure 2:**
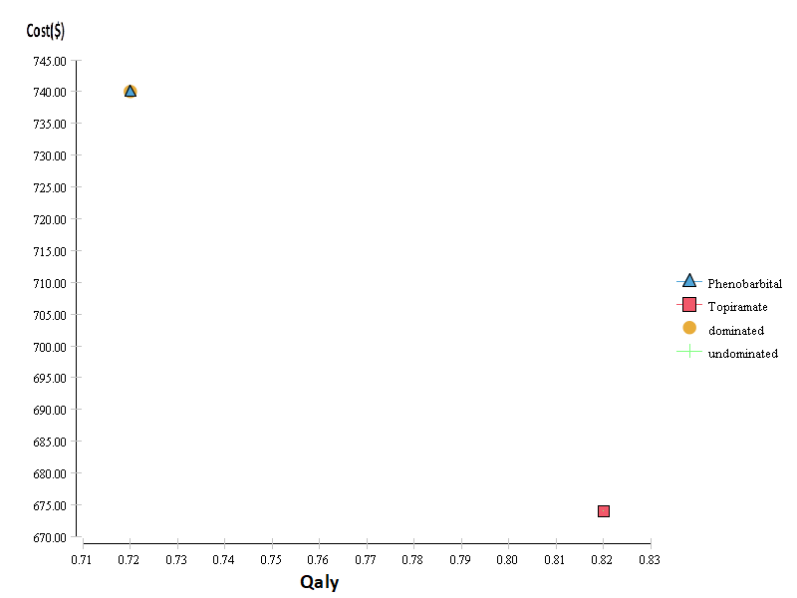
Cost-Utility analysis of the use of phenobarbital and topiramate for treating patients less than five years of age with febrile seizure

As presented in Figures 1, 2 and Table 3, the results of cost-effectiveness analysis and cost-utility analysis showed that the calculated effectiveness of phenobarbital and topiramate, without the expected side effects, was 0.3 and 0.63 (with two decimal places), respectively; also, the scores of quality of life were 0.72 and 0.82, and the expected costs were $740 and $642, respectively. Therefore, topiramate had a lower cost, was more effective, and had a better level of utility than phenobarbital. Thus, it is more preferable than phenobarbital. Table 3 presents the findings on cost, effectiveness, incremental cost, incremental effectiveness, incremental cost-effectiveness ratio (ICER), as well as the dominance of drugs over each other in the two groups of patients with febrile seizure. As compared with topiramate, phenobarbital caused more febrile seizure, had lower effectiveness, provided a lower quality of life score, and imposed additional costs on the patient. Thus, topiramate had the lowest cost, the highest expected utility, the highest level of effectiveness, with no recurrence of the expected febrile seizure within a six-month period of follow up.

**Uncertainty analysis**

The effects of uncertainty were studied using one-way sensitivity analysis and the values of each variable changed by 20% plotted in the form of a tornado diagram.

**Figure 3:**
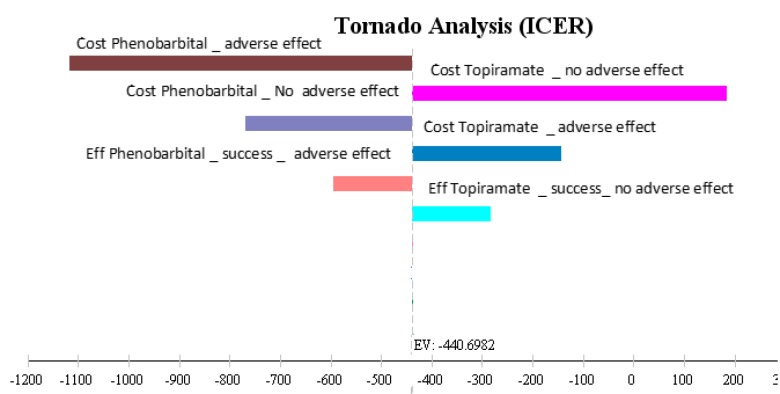
Tornado diagram of cost-effectiveness for patients with febrile seizure treated with phenobarbital and topiramate

Figure 3 presents the results of one-way sensitivity analysis in the form of a tornado diagram. Changes in many parameters did not have much effect on the results of the study. However, incremental cost-effectiveness ratio had the highest level of sensitivity to the cost of phenobarbital in patients with complications and had the lowest level of sensitivity to the likelihood of treatment failure of topiramate in patients without complications. In fact, the price of phenobarbital in patients with complications of the disease was a decisive parameter in the ICER.

**Figure 4:**
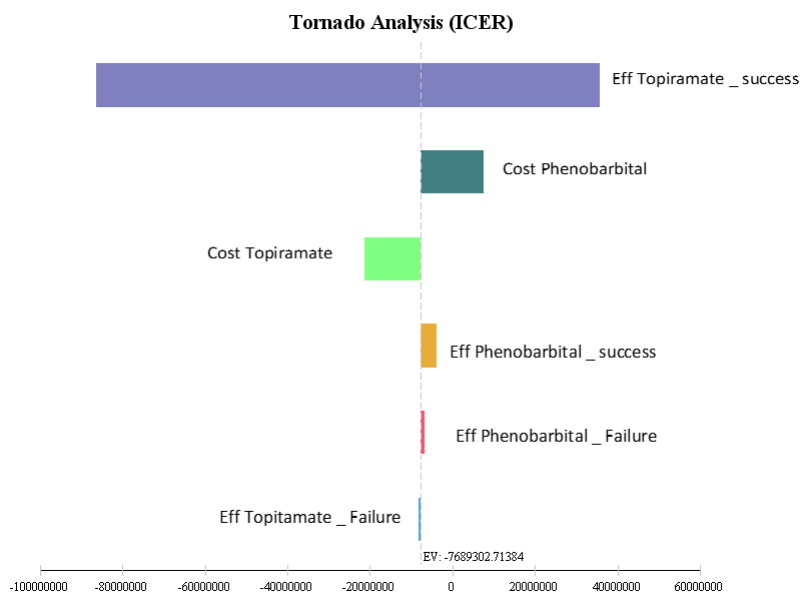
Tornado diagram of cost-utility for patients with febrile seizure treated with phenobarbital and topiramate

The results of the tornado diagram indicate that incremental cost-effectiveness ratio had the highest level of sensitivity to the utility of topiramate and had the lowest level of sensitivity to the utility of the phenobarbital. In fact, the likelihood of the effectiveness of topiramate was the essential parameter in the ICER (Figure 4).

**Table 1:** Relative and absolute frequency of demographic characteristics of children less than five years of age with febrile seizure in 2016-2017

**Variable**	**Type**	**Number**	**Percentage**
Province of residence	Fars	80	87.9
	Other provinces	11	12.1
Place of residence	City	62	68.1
	Village	29	31.9
Sex	Female	31	34.1
	Male	60	65.9
Type of febrile seizure	Simple	51	57.1
	Complex	38	42.9
The number of patients with simple febrile seizure	Topiramate	23	45.1
	Phenobarbital	28	54.9
The number of patients with complex febrile seizure	Topiramate	17	44.8
	Phenobarbital	21	55.2
Family history of febrile seizure	Yes	33	36.2
	No	58	63.8
Type of drug used	Topiramate	40	43.9
	Phenobarbital	51	56.1
Recurrence rate of FC in two groups	Yes	22	24.2
	No	69	75.8

**Table 2:** Mean direct and indirect costs of treatment using phenobarbital and topiramate in children less than five years of age with febrile seizure

Type of service	Phenobarbital			Topiramate		
	PPP$	Percentage	Standard deviation	PPP$	Percentage	Standard deviation
**Direct medical costs**						
Medication	20.09	2.9	13	44.88	7	.30
Visits to the doctor	93.1	11.55	54	80.9	11	46
Laboratory tests and Diagnostic services	193.11	23.98	100	188.6	25.9	109
Hospitalization	16.29	2.3	10	0	0	0
Total	322.59	41.73	73	314.38	44	75
**Direct non-medical cost**						
Transportation	145.73	20.4	91	128.61	20.01	64
Accommodation	82.55	11.55	51	77.16	12.01	50
Meals	55.03	7.7	30	51.44	8	34
Total	283.32	39.65	66	257.23	40.03	62
**Indirect Costs**	133	18.61	83	103.05	16.04	54
Total	739.3	100	448	674	100	421

**Table 3:** Results of cost-effectiveness and cost-utility analysis of the use of phenobarbital and topiramate for treating patients with febrile seizure

Strategy	Cost	Utility	Eff	Incremental cost	Incremental effectiveness	Incremental utility	ICER (cost-effectiveness)	ICER(cost-utility)	Subset
Topiramate	673	0.82	0.64	0	0	0	need to calculate ICER	No need to calculate ICER	Dominant
Phonobarbital	740	0.72	0.3	66.82	-0.32	-0.11			Dominated

## Discussion

The aim of this study was to evaluate the cost-effectiveness of phenobarbital and topiramate drugs in patients with febrile seizure. Phenobarbital is commonly prescribed in Iran for children with febrile seizure, but in our county, topiramate is not prescribed for children with febrile seizure and is only prescribed for epileptic patients. However, the pilot administration of topiramate for children with febrile seizure has recently been started. The aim of this study was to examine the cost-effectiveness of these two drugs in order to choose the best treatment.

Since no specific study has been conducted on ​​febrile seizure so far, we have compared our findings with the results of the studies conducted on patients with epilepsy, which is a long-term, progressive, and persistent type of febrile seizure. Most drugs used for patients with epilepsy are similar to those used for treating febrile seizure (25).

In comparison with phenobarbital, topiramate was a better option in terms of both cost-effectiveness and cost-utility. As to the lack of recurrence of febrile seizure in both groups, the results of ICER showed that among treatment strategies in the country, topiramate was a superior and cost-effective option because of its higher efficacy and lower costs than phenobarbital. The results of this study are consistent with those of a study (26) suggesting topiramate was a cost-effective drug.

Moreover, as to the utility outcome in both groups, the results of ICER showed that among treatment strategies in the country, topiramate was superior in terms of utility and had a lower cost; thus, it was more cost-effective than phenobarbital. The results of this study are consistent with a study (27) that reported topiramate as a drug with a high level of cost-utility. Nevertheless, the results of this study were not consistent with another study (28), showing that topiramate is not a drug with a higher level of cost-utility.

Furthermore, the results of one-way sensitivity analysis showed that the incremental cost-effectiveness ratio had the highest level of sensitivity to the "utility of patients who consumed topiramate", and incremental cost-effectiveness ratio had the highest level of sensitivity to "the cost spent by patients who consumed phenobarbital". In both cases, the ICER value was negative; it is possible to make definite conclusions about the results of the study. Therefore, sensitivity analysis did not change the status of topiramate as the most effective drug; it is a sign of the robustness of the results of the study. Therefore, the results of the present study are consistent with other findings (26, 27).

The results of this study showed that when the outcomes of the econometric evaluation are utility and the lack of recurrent febrile seizure in children with the disease, topiramate drug is more cost-effective than the phenobarbital and it is more cost-effective, favorable, and the dominant option because phenobarbital has a higher expected cost and a lower efficacy and lower utility than topiramate.

Among the strengths of this study, we can mention the followings: inclusion of all costs, including direct medical and non-medical costs, as well as indirect costs in the model, and the use of regional data on costs and effectiveness collected from the self-reports of the patients.

## Limitations

The study had some limitations. The number of patients in this study was small. Conducting the study over a longer period could help to show the relationships better. Of course, there was no problem in finding the patients receiving phenobarbital because it is a common drug that physicians routinely prescribe for the patients, but we had problem finding patients and obtaining their parents’ consent to receive topiramate over the first two months and even some patients quitted the study (this issue was also investigated and they quitted the study not because of the drug side effects, but because in their viewpoint topiramate was not a common drug for treating febrile seizure and they had some concerns about this issue). Moreover, one of the limitations in this study is that the types of complex FC (focal, recurrent or prolonged) are not mentioned in two groups, and may effect on recurrence rate. 

In addition, topiramate was the most effective and superior option for treating the patients, it is necessary to generalize the results of this study to other settings; for instance, it is necessary to consider and assess the epidemiology of the disease and demographic structure, availability of the resources, costs, evaluation of the outcomes by individuals, threshold, and use of various indicators of effectiveness in various studies that may affect the outcomes of this study. Since the samples in the present study were mainly from the south of the country, we should be cautious in generalizing the results to other communities.

**In conclusion, **topiramate in children under five years of age with febrile seizure is a superior strategy with high-cost effectiveness and can be considered as a high priority drug, as compared with phenobarbital. Moreover, its use as the first line of treatment reduces the time of treatment and the cost of drug resistance, as compared with the phenobarbital drug. Hence, in order to reduce the burden of the disease in the community, topiramate should be used as the first line of treatment in children under five years of age with febrile seizure.

## Author`s Contribution

Hamid Nemati: Participated in design of study, supervised whole study, and revised the paper critically for important intellectual content; Hamid Talebianpour: participated in design of the study, the acquisition, analysis, and interpretation of the data. Farhad Lotfi: Drafting of the manuscript, revised the paper critically for important intellectual content. Nazanin Zahra Sepehri: Acquisition of data and statistical analysis of data. Khosro Keshavarz: Participated in design of study and drafting of the manuscript, analysis, interpretation of the data, and final revision of the manuscript. All authors read and approved the final manuscript.

## Conflict of interest

The authors declare that there is no conflict of interests.
